# A methylprednisolone-loaded and core-shell nanofiber-covered stent-graft to prevent inflammation and reduce degradation in aortic dissection

**DOI:** 10.1186/s40824-022-00259-5

**Published:** 2022-04-25

**Authors:** Junjun Liu, Hongqiao Zhu, Yifei Pei, Heng Zhang, Jian Zhou, Zaiping Jing

**Affiliations:** 1 Department of Vascular Surgery, the Affiliated Hospital of Qingdao University, Qingdao, Shandong 266003 China; 2 Department of Vascular Surgery, the First Affiliated Hospital of the Navy Medical University, Shanghai, 200433 China

**Keywords:** Coaxial nanofiber, Methylprednisolone, Inflammation, Extracellular matrix degradation, Aortic dissection

## Abstract

**Background:**

Stent-graft-induced inflammation is an independent risk factor for adverse aortic remodeling in aortic dissection. In this context, we asked that whether a methylprednisolone-loaded stent-graft could reduce inflammation and degradation.

**Methods:**

First, a coaxial electrospinning technique was used to create a core-shell film with methylprednisolone encapsulated in the inner of poly (L-lactide-co-caprolactone) nanofibers for controllable drug release. Second, an in vitro study was conducted to evaluate the biocompatibility of the nanofiber meshes. Third, the porcine aortic dissection model was developed to investigate the therapeutic effects of the methylprednisolone-loaded stent-graft.

**Results:**

The results demonstrated that the nanofiber-coated film with a methylprednisolone-poly-caprolactone core layer and a poly (L-lactide-co-caprolactone) shell layer could effectively sustain drug release in vitro. In vivo study showed that the methylprednisolone-loaded stent-graft could reduce degradtion of aortic dissection by regulating inflammation.

**Conclusions:**

Overall, the controllable drug release by coaxial nanofiber is a promising approach to alleviate aortic inflammation and promote aortic remodeling after stent-graft implantation.

**Supplementary Information:**

The online version contains supplementary material available at 10.1186/s40824-022-00259-5.

## Introduction

Aortic dissection (AD) is one of the most devastating aortic diseases, which is caused by an entry tear in the aortic intima or hemorrhage in the aortic media, leading to the separation of the aortic layers [1, 2]. According to the Stanford classification, type B aortic dissection (TBAD) originates from the distal to the ostium of the left subclavian artery [3]. In recent years, thoracic endovascular aortic repair (TEVAR) for TBAD is recommended by more and more surgeons because of long-term higher survival rates and more positive aortic remodeling compared with medical treatment [4]. Despite the promising potential of TEVAR on TBAD, the stent-graft-related adverse events cannot be ignored. According to the meta-analysis by us, the pooled overall incidence of re-intervention was estimated to be 8.1–15% after TEVAR [1].

There has recently been a significant body of published work indicating that perioperative high inflammatory status is a strong predictor of adverse early and late complications in patients after TEVAR [5, 6]. Furthermore, higher ^18^F-fluorodeoxyglucose (^18^F-FDG) uptake in the dissected aortic wall was confirmed to independently predict progressive aortic dilation after TEVAR [7, 8]. This evidence suggests that persistent inflammatory conditions on the aortic dissecting wall may lead to fragility and rupture [9]. Recently, our studies proved that endothelial cell dysfunction, which was triggered by inflammation, barrier dysfunction, and blood clotting, ultimately leads to aortic adverse remodeling [10, 11]. However, few studies focus on inflammation prevention and endothelial protection in the aortic wall after TEVAR.

In a previous study, we discovered that glucocorticoids could reduce tumor necrosis factor-α (TNF-α) secretion while increasing uncombined TNF-soluble receptor II (TNF-sRII) in the aortic dissection, thus positively promoting aortic remodeling [12]. Furthermore, the efficacy and safety of intravenous glucocorticoid administration after TEVAR were conducted in our center [13]. The results showed that postoperative dexamethasone delivery could improve the postoperative symptoms after the operation. However, no difference was observed in a 3-month follow-up of aortic remodeling or all-cause mortality [13]. It suggested that the anti-inflammatory effect of intravenous glucocorticoid administration was for a short duration. In this research context, we asked whether controllable methylprednisolone-loaded (MPSS-loaded) film fabricated on a stent-graft could reduce leukocyte activation, enhance reendothelialization, and prevent extracellular matrix degradation in the aortic dissecting wall.

In this study, we constructed a core-shell nanofiber-coated stent-graft to sustain controllable drug release of MPSS. First, we characterized the in vitro features of the MPSS-loaded mesh films. Second, we performed an in vitro experiment to examine the cell proliferation and anti-inflammatory effects of the MPSS-loaded mesh films. Third, we evaluated the in vivo therapeutic effects of MPSS-loaded stent-grafts implanted in porcine aortic dissection models.

## Materials and methods

### Materials

Polycaprolactone (PCL) 80 K (Mw =80 kDa) and poly (L-lactide-co-caprolactone) (PLCL) with an L-lactic acid/ε-caprolactone ratio of 75:50 (MW =385 kDa) were from Patos Biomaterial Co., Ltd. (Tianjin, China). Chromatographic (chromatographical purity) was bought from Fisher Scientific (Leicestershire, UK). Phosphoric acid was purchased from Sinopharm Group Chemical Reagent Co., Ltd. Methylprednisolone sodium succinate (MPSS, purity ≥97%) was purchased from Shanghai Haling Biotechnology Co., Ltd., Shanghai, China. Lipopolysaccharides (LPS) were purchased from Medchemexpress, Shanghai, China. All other chemicals were of analytical grade and used as received. All the deionized water was homemade. The Gore Excluder Iliac Branch Endoprosthesis was kindly provided by the Gore Company (IBE, W. L. Gore and Associates, Flagstaff, AZ, USA).

### Film preparation

**Fig. 1:**
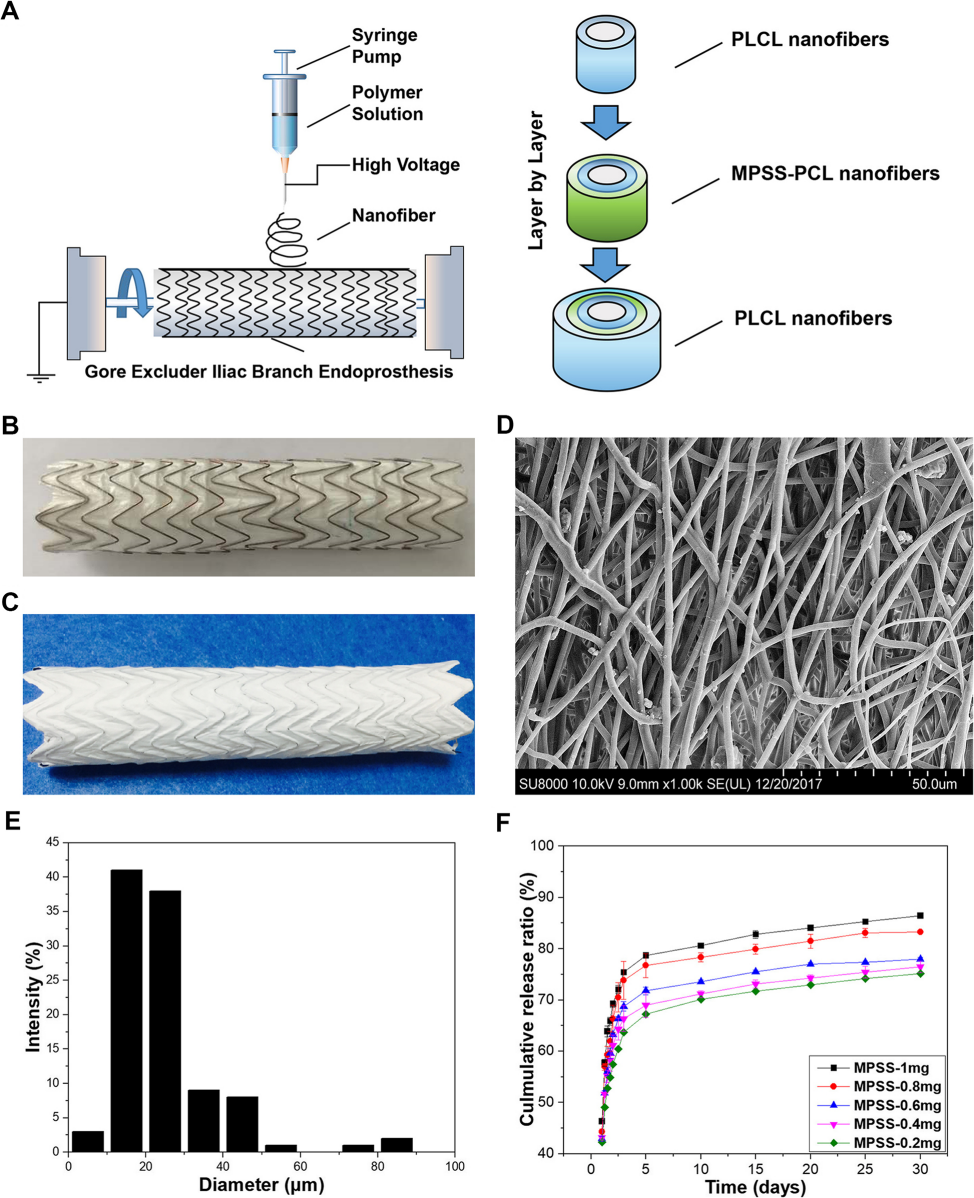
Fabrication of the drug-loaded stent-graft. **A** The illustration of a sandwich-like film with a coaxial electrospinning technique was developed, in which MPSS was encapsulated in the inner of PLCL nanofibers; **B** The Gore Excluder Iliac Branch Endoprosthesis; **C** The Gore Excluder Iliac Branch Endoprosthesis covered with the MPSS-loaded film; **D** SEM micrographs of electrospun nanofibers at a magnification of 1000 (scale bar = 50 μm). **E** Diameter distribution of electrospun nanofibers; **F** In vitro release behaviors of MPSS-loaded films with 0.2 mg, 0.4 mg, 0.6 mg, 0.8 mg and 1 mg

For the controllable drug release, a core-shell film with a coaxial electrospinning technique was developed, in which MPSS was encapsulated in the inner PLCL nanofibers (Fig. 1A). The composition of the drug layer was composited by PCL and MPSS. The non-drug shell layer was formed by PLCL. A PCL solution was prepared using N, N-dimethylformamide as a solvent (the concentration was 10%). Different doses of MPSS were dissolved (0.2 mg, 0.4 mg, 0.6 mg, 0.8 mg and 1 mg). A PLCL solution was prepared utilizing hexafluoroisopropanol as a solvent and the concentration was 10%.

### Covered stent-grafts

Stent-grafts were manufactured by coating the MPSS-loaded or non-MPSS-loaded film onto a stent graft (Gore, Excluder, 18 mm). Figure 1A demonstrates that the electrospinning emulsion solution being ejected through a modified needle attached to a high voltage DC power supply (receiving distance = 10–15 cm, solution flow rate = 10 μl/min, DW-DP503/303-1ACF1). During the electrospinning process, a positive voltage of 10–15 KV was applied to the needle. The nanofibers were collected on the film of the Excluder stent-graft or aluminum coils. The experiment conditions were set at 25 °C and 20% humidity. The collected nanofiber meshes received vacuum drying and sterilization before the in vitro and in vivo evaluations. The emulsions with 0.2, 0.4, 0.6, 0.8, and 1 mg of MPSS layer-by-layer nanofiber mesh were identified as MPSS-0.2 mg, MPSS-0.4 mg, MPSS-0.6 mg, MPSS-0.8 mg and MPSS-1 mg, respectively. The meshes created without MPSS were identified as PLCL-N. The nanofiber mesh-covered stents were fabricated by electrospinning MPSS-1 mg emulsions onto the Gore Excluder stent-grafts.

### Scanning electron microscopy

Nanofiber mesh samples were cut into 0.5 × 0.5 cm^2^ squares, then fixed on the sample table with double-sided tape, and sprayed with gold by the ion sputterer (IB-3, Eiko Engineering, Hitachinaka, Japan) for 3 min. Then the prepared samples were imaged by a scanning electron microscope (SEM) Hitachi SU8000 (Hitachi, Minato-ku, Tokyo, Japan). The fiber diameter distribution was measured by the Image J software at 1000× magnification (Windows Edition, National Institutes of Health, Maryland, USA).

### In vitro release

In the in vitro release experiments, the nanofiber mesh films were cut into 1 × 1 cm^2^ squares and put into a polyethylene tube, which contains pH 7.4 phosphate buffer solution (PBS). After that, the tubes were put into a 37 °C constant temperature water bath and stirred at a speed of 100 rpm. According to the pre-determined schedule, the medium was replaced with fresh PBS and the withdrawn medium was evaluated by high-performance liquid chromatography (HPLC).

### Cell proliferation

The medium of human umbilical vein endothelial cells (HUVECs) was RPMI-1640 containing 10% fetal bovine serum and 1% antibiotic antimycin (penicillin and streptomycin: 100 units/ml). HUVECs were then cultured in an incubator at 37 °C with 5% CO_2_. One in vitro experiment was conducted in the 24-well plates, in which the nanofiber samples were placed in advance to adjust the pH value of the cell culture medium. The HUVECs were treated with different types of nanofiber samples when the HUVEC density reached 1 × 10^4^ cells/well. After 24h-culturing, each well was supplemented with 100ul of CCK-8 reagent and the cells were further incubated at 37 ℃.The optical density (OD) values at 450 nm were measured with a microplate reader (INFINITE M NANO, Tecan, California, USA) after culture for an additional 2 h.

### Cell migration

The in vitro scratch wound assay was a well-developed method to measure cell migration. HUVECs were cultured in the 6-well plates and preincubated with nanofibers for 2 days. A micropipette tip was utilized to wound the cell cluster with a scratch, then the cells were rinsed with PBS and incubated at 37 ℃ for 24 h. At 0 h, 6 h, and 24 h after the procedure, the scratching closure was imaged with a digital camera. The ImageJ program (National Institutes of Health, Bethesda, MD, USA) was used to make the measurement and calculate the ability of cell migration in a time-dependent manner.

### In vivo examination

A porcine aortic dissection model was developed to evaluate the safety and efficacy of the MPSS-loaded nanofiber stent-graft. All animal operations were in accordance with the Animal Management Rules of the Ministry of Health of the People’s Republic of China (No. 55, 2001). The in vivo experiment was approved by the Ethics Committee of the First Affiliated Hospital to Navy Medical University (CHEC-Y2020–042, April 21, 2020).

### Porcine model of aortic dissection

The pig was placed under general anesthesia through a ventilator following an intravenous induction of anesthesia. Intravenous antibiotics were supplied prophylactically prior to surgery and heparin (50 IU/kg) was administered intravenously to ensure that the activated clotting time during operation was more than 300 s. The left lobe of the lung was retracted and the descending aorta was exposed by conducting a thoracotomy via the sixth intercostal gap on the left side and extending the upper border of the rib cage. The descending aorta was separated bluntly for approximately 5–10 cm (Supplemental Fig. 1A), and the free section of the intercostal artery was ligated. The outer layer of the aorta was carefully incised transversely with a sharp knife to a depth of approximately 0.5 mm, approximately 1/3 of the wall thickness, and approximately 3 mm in length, and the intercalated lumen was gently separated caudally with a mosquito clamp to a length of approximately 5 mm and a width of approximately 1/2 the tube diameter (Supplemental Fig. 1B). The lumen was split by pressing down along the lumen with a handmade lamellar separator to a length of approximately 10 cm. A 20 ml syringe was linked to an infusion tube and put in the isolated the false lumen, and heparin saline was injected in a pulsatile way to expand the false lumen by simulating the pulsating blood flow (Supplemental Fig. 1C). The presence of regurgitant bleeding indicated the appearance of a distal tear in the aorta, then the injection stopped. The aortic incision was anastomosed with continuous 5–0 prolene polypropylene monofilament sutures, and the entire wall of the head end was anastomosed with the caudal outer membrane (Supplemental Fig. 1D). After completion of suturing, vascular ultrasonography was performed to confirm successful aortic coarctation stenting (Supplemental Fig. 1E).

### Stent-graft implantation

A total of 20 pigs were randomly allocated into the sham group (*n* = 5), the AD model group (*n* = 5), the TEVAR group (*n* = 5) or the 1 mg-MPSS-loaded TEVAR (MPSS-TEVAR) group (*n* = 5). Thoracotomy and aorta mobilization was done in the sham group; the AD modeling operations were done as described in the AD model group, the TEVAR group and the MPSS-TEVAR group; stent-graft implantation was done 3 days after AD modeling in the TEVAR group and the MPSS-TEVAR group (Supplemental Fig. 1F-H). After implantation, digital subtraction angiography (DSA) was performed. The CTA was conducted immediately, one week and one month after the implantation.

### In vitro and in vivo cytokine levels

An in vitro cell experiment was performed to examine the anti-inflammatory effects of MPSS-loaded films. HUVECs were seeded in a 24-well plate and cultivated until reaching 80–90% confluency; subsequently, cells were pretreated with 100 ng/ml LPS for 12 h, followed by treatment with various dosages (0.2 mg, 0.4 mg, 0.6 mg, 0.8 mg, and 1 mg) of MPSS-loaded films for an additional 12 h. The evaluated samples were from the porcine serum in each group.

The pro-inflammatory cytokine levels of TNF-α, interleukin-6 (IL-6) and C-reactive protein (CRP) in porcine serum, and TNF-α and IL-6 in the HUVEC-conditioned medium were measured using enzyme-linked immunosorbent assay (ELISA) quantification kits (R&D Systems, MN, USA). The absorbance valves at a wavelength of 450 nm were measured with a microplate reader (INFINITE M NANO, Tecan, California, USA).

### PET/CT imaging

To minimize the effect of blood retention on aortic dissection pathologies uptake, a dynamic PET scan and corresponding CT images were obtained 45 to 60 min after a tail vein injection of ^18^F-FDG (3.70–5.55 MBq/kg) using PET/CT (Biograph TruPoint, Siemens, Malvern, PA, Germany). The CT scanning parameters were set as follows: scanning time: 18.7–21.9 s; slice thickness: 3 mm; pitch: 1.2; gantry speed: 0.5 s/rotation; tube current: 140 mAs; tube voltage: 120 kVp. Image reconstruction was performed using an ordered subset expectation maximization (OSEM) algorithm.

^18^F-FDG uptake was measured in the aortic wall and inferior vena cava wall [defined as the maximum and mean standardized uptake value (SUVmax and SUVmean, respectively)] approximately each 5 mm on axial images. Thereafter, the target-to-background ratio (TBR) was obtained (SUVmax/SUVmean ratio).

### Histopathology and immunohistochemistry

On day 30 after stent-graft implantation, the descending aorta were harvested from pigs in each group (Supplemental Fig. 1H). For histological analysis, dissected samples were fixed in 4% paraformaldehyde for 48 h. According to the standard methods, the 5 μm sections from each group were stained with Hematoxylin and eosin (H&E), Masson trichrome and elastic-van Gieson staining (EVG). Tissues were also immunostained with rabbit MPO antibody (1: 200, A0398, DAKO); rabbit CD68 antibody (1: 200,ab125212, abcam); rabbit ICAM-1 antibody (bs-4617R,Bioss); rabbit VCAM-1 antibody (bs-0920R, Bioss); rabbit P-selectin antibody (bs-0561R, Bioss). The images of sections were recorded with a light microscope (Leica, Wetzlar, Germany).

Vessel inflammation was rated on a scale of 0 to 3 according to previously published methodology [14]. The grade of elastin degradation was according to previous studies: grade 1, intact, well-organized elastic laminae; grade 2, elastic laminae with some interruptions and breaks; grade 3, severe elastin fragmentation or loss [15]. Masson trichrome was utilized to assess the extracellular matrix collagen deposition [16].

All immunohistochemistry staining was quantified as the area of positive staining expressed as a percentage of the total neointimal area.

### Western blot analysis

Proteins were extracted from the harvested aorta samples using cell lysis buffer (P0013C, Beyotime, Shanghai, China). The following antibodies were used: β-actin antibody (1: 10000, 66,009–1-Ig, Proteintech); rabbit ICAM-1 antibody (1: 200, bs-4617R,Bioss); rabbit VCAM-1 antibody (1: 200, bs-0920R, Bioss) and rabbit P-selectin antibody (1: 200, bs-0561R, Bioss). Then, the membranes were incubated with the corresponding secondary antibodies. The blots were visualized using a chemiluminescence reagent. The quantification of the Western blot bands was performed using ImageJ software (National Institutes of Health, Bethesda, MD, USA).

### Statistical analysis

The presentation of continuous variables was determined to be mean ± standard deviation. The difference between the groups was compared by utilizing the one-way analysis of variance (ANOVA). All statistical analyses were performed using SPSS software (version 26.0; SPSS Inc., Chicago, IL, USA). All the tests were 2-sided and *p* < 0.05 was considered to be statistically significant.

## Results

### Characteristics of the nanofiber meshes

Figure 1B and C show the Gore Excluder Iliac Branch Endoprosthesis uncovered or covered with the PLCL-MPSS film. Figure 1D demonstrates that, in the SEM micrography, the nanofibers are smooth and continuous. The diameter distribution of the nanofibers is shown in Fig. 1E and the diameter of the nanofibers is estimated to be 15–20 μm. The PLCL-MPSS film in vitro release curves obtained for the time points evaluated are depicted in Fig. 1F. In the first 5 days, the cumulative release from MPSS-0.2 mg is 67.2%, MPSS-0.4 mg is 69.0%, MPSS-0.6 mg is 71.7%, MPSS-0.8 mg is 76.7%, and from the MPSS-1 mg is 78.6%. The release of MPSS in each group is followed by a sustained high release the next 25 days, in which the cumulative release of MPSS-0.2 mg is 75.1%, MPSS-0.4 mg is 76.4%, MPSS-0.6 mg is 77.1%, MPSS-0.8 mg is 83.2%, and from theMPSS-1 mg is 86.4%.

### Cell proliferation, migration and in vitro inflammation evaluation

**Fig. 2:**
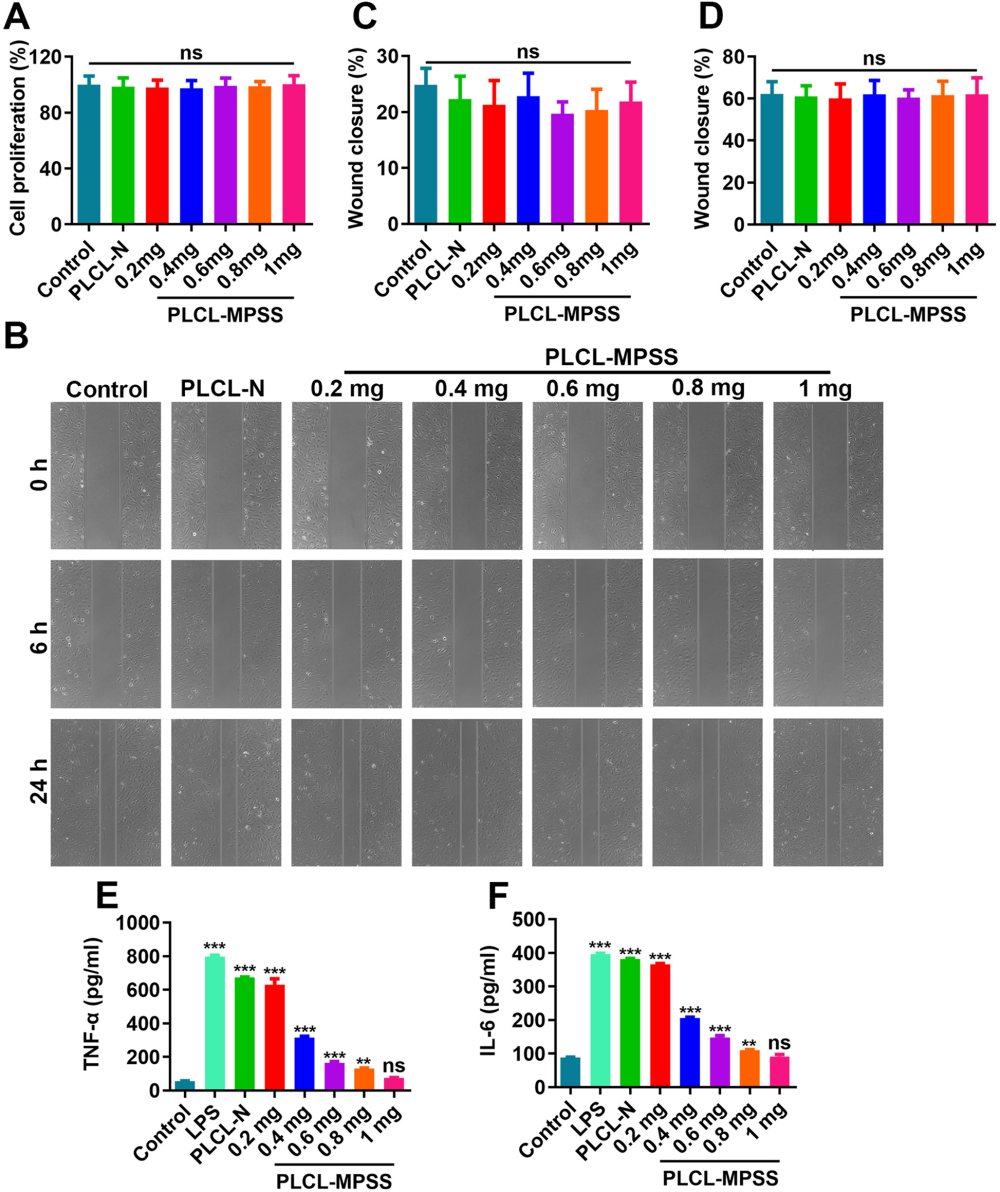
In vitro cell proliferation, migration and inflammation tests on each nanofiber mesh. **A** HUVECs were treated with PLCL-N, MPSS-loaded with 0.2 mg, 0.4 mg, 0.6 mg, 0.8 mg and 1 mg for 24 h, the optical density (OD) values at 450 nm were measured and the cell proliferation was determined by CCK-8 assay; **B** The migration was determined by scratch wound assay after co-culture for 48 h and monitored through the use of digital photography at time of 0 h, 6 h and 24 h; **C** Rates of wound closure in each group at time of 6 h; **D** Rates of wound closure in each group at time of 24 h. **E**, **F** HUVECs were induced with LPS 100 ng/ml for 24 h, then treated with PLCL-N, MPSS-loaded with 0.2 mg, 0.4 mg, 0.6 mg, 0.8 mg and 1 mg for another 24 h. The supernatant of each group was collected to test the levels of tumor necrosis factor-α (TNF-α, E) and interluekin-6 (IL-6, F) by ELISA. The data are expressed as mean ± SD. Statistical significance:** *P* < 0.01 vs. control group; *** *P* < 0.001 vs. control group; ns *P* > 0.05 vs. control group. Ns indicates no significance

To assess the cytocompatibility of PLCL-MPSS film, cell proliferation assay of HUVECs was performed. The CCK-8 assay shows that the proliferation of HUVECs incubated with PLCL, PLCL-MPSS with 0.2 mg, 0.4 mg, 0.6 mg, 0.8 mg and 1 mg have no obvious difference (Fig. 2A).

Scratch wound assay is also performed to evaluate the regulation of PLCL-MPSS film meshes on HUVECs migration (Fig. 2B). We found that there was no obvious difference among the groups in 6 h and 24 h (Figs. 2C, D). These results indicates that the examined films have no significant toxicity against HUVECs.

To evaluate the optimal concentration of MPSS, the inflammatory cytokine levels were compared in each group. Results show the levels of tumor necrosis factor-α (TNF-α, Fig. 2E) and interluekin-6 (IL-6, Fig. 2F) in HUVECs treated with MPSS-1 mg mesh film are similar with the control group (*P* > 0.05).

### In vivo examination

**Fig. 3:**
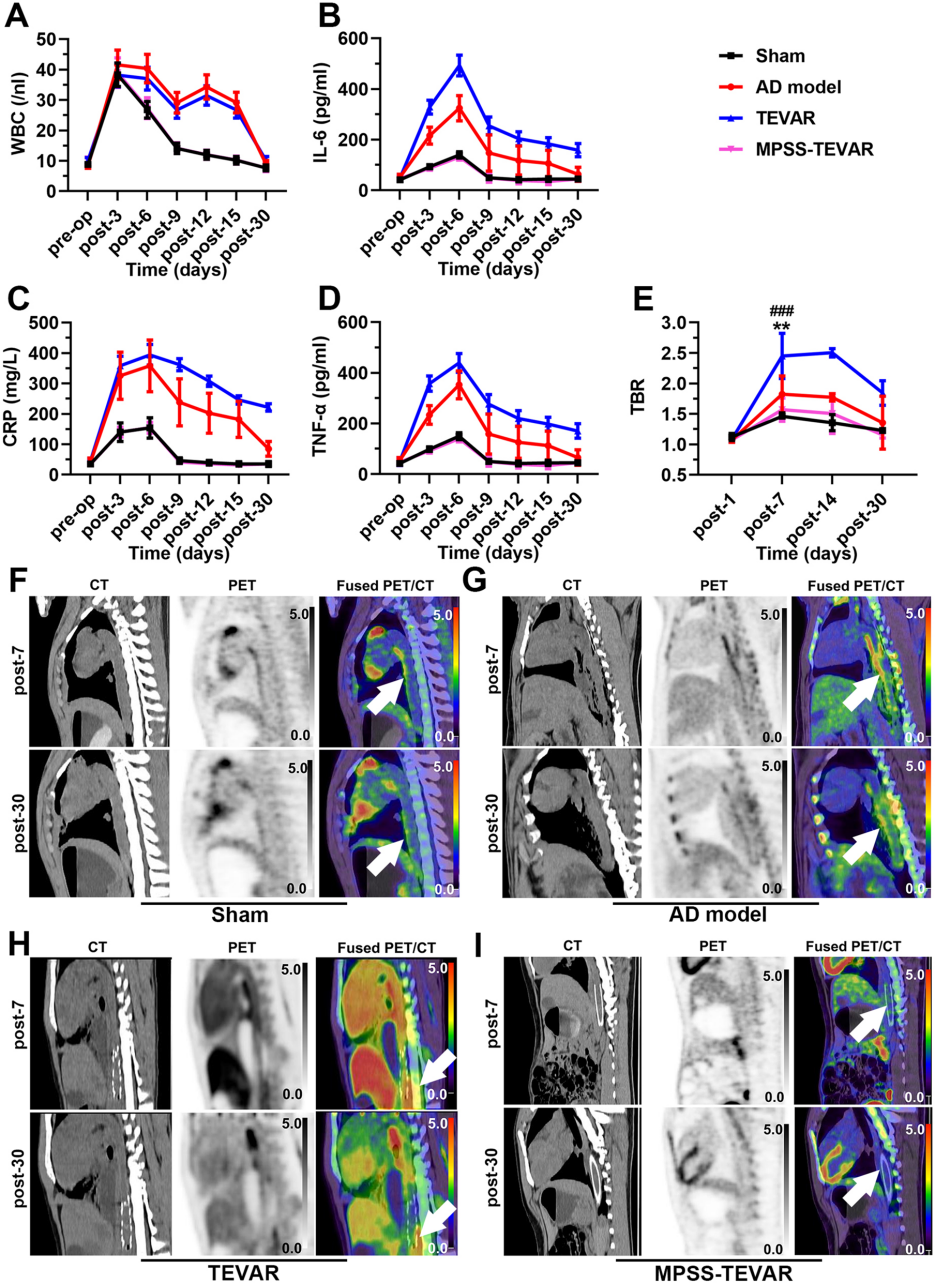
In vivo examination of MPSS-loaded stent-grafts in porcine AD model. **A**-**D** Time course of serum levels of white blood cells WBC (**A**), IL-6 (**B**), CRP (**C**) and TNF-α (**D**); **E** TBR values for each group across the imaging time points; **F** CT, PET and fused PET/CT images of porcine aorta 7 and 30 days after operation in the sham group, white arrows show the location of SUVmax. **G** CT, PET and fused PET/CT images of porcine aorta 7 and 30 days after operation in the AD model group, white arrows show the location of SUVmax; **H** CT, PET and fused PET/CT images of porcine aorta 7 and 30 days after operation in the TEVAR group, white arrows show the location of SUVmax; **I** CT, PET and fused PET/CT images of porcine aorta 7 and 30 days after operation in the MPSS-TEVAR group, white arrows show the location of SUVmax. The data are expressed as mean ± SD. Statistical significance:^**^P<0.01, TEVAR vs. AD group; ^###^*P*<0.001, TEVAR vs. MPSS-TEVAR group. WBC, white blood cells; IL-6, interluekin-6; CRP, C-reactive protein; TNF-α, tumor necrosis factor-α; TBR, tissue-to- background ratio

Figure 3A-D demonstrates the systemic inflammatory responses observed in each group. More precisely, there is a significant increase compared with the baseline values in each group, of WBC, IL-6, CRP and TNF-α. Moreover, by comparing biomarker peak values during observation between the TEVAR and MPSS-TEVAR groups, we found that the TEVAR group featured a more pronounced and enduring systemic inflammatory response than the MPSS-TEVAR group over the 30-day observation period.

The statistical results show TBR values increase significantly 30 days after AD modeling in all groups (Fig. 3E). Quantitatively, peak value of TBR in TEVAR group is significantly higher than AD group and MPSS-TEVAR group (*P* = 0.006 and *P* < 0.001 respectively). Figure 3F shows there is no obvious increased ^18^F-FDG uptake in the location of aorta in the sham group. PET-CT imaging shows significantly increased ^18^F-FDG uptake in the dissecting aortic wall 7 days after modeling and residual ^18^F-FDG uptake 30 days after modeling (Fig. 3G and H). Figure 3I demonstrates that there is no obvious increased ^18^F-FDG uptake 7 days and 30 days after MPSS-loaded stent-graft implantation.

### Histologic examination

**Fig. 4:**
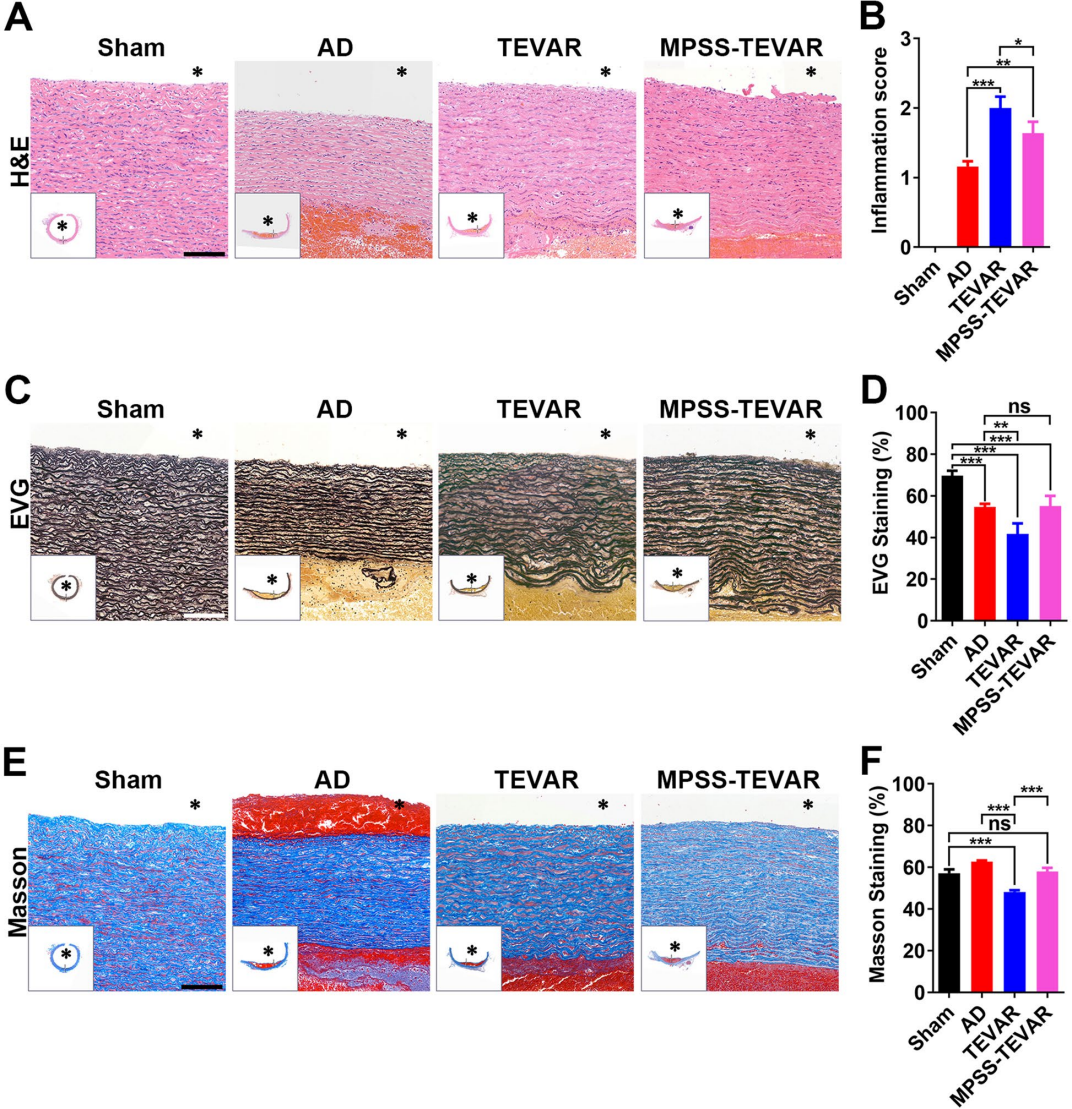
MPSS-loaded stent-grafts reduced inflammation and alleviated extracellular matrix degradation in AD model. **A** H&E staining was performed in each group, bar =100 μm; **B** Inflammation scores were compared among groups; **C** EVG staining was performed in each group, bar =100 μm; **D** Elastic fiber area of media area (%) was compared among groups; **E** Masson staining was performed in each group, bar =100 μm; **F** Collagen area of media area (%) was compared among groups. The data are expressed as mean ± SD. Statistical significance:. * *P* < 0.05; ** *P* < 0.01; *** *P* < 0.001. Ns indicates no significance

The changes in the structure of the aortic wall mainly manifested the infiltration of inflammatory cells (Fig. 4A). Inflammation scores were significantly elevated in the TEVAR groups compared with the AD group (*P* < 0.001) and the inflammation was relieved in MPSS-TEVAR compared with the TEVAR group (*P* < 0.05, Fig. 4B). Figures 4C and E show the representative images of EVG, and Masson staining in each group.

### Immunohistochemistry evaluation

**Fig. 5:**
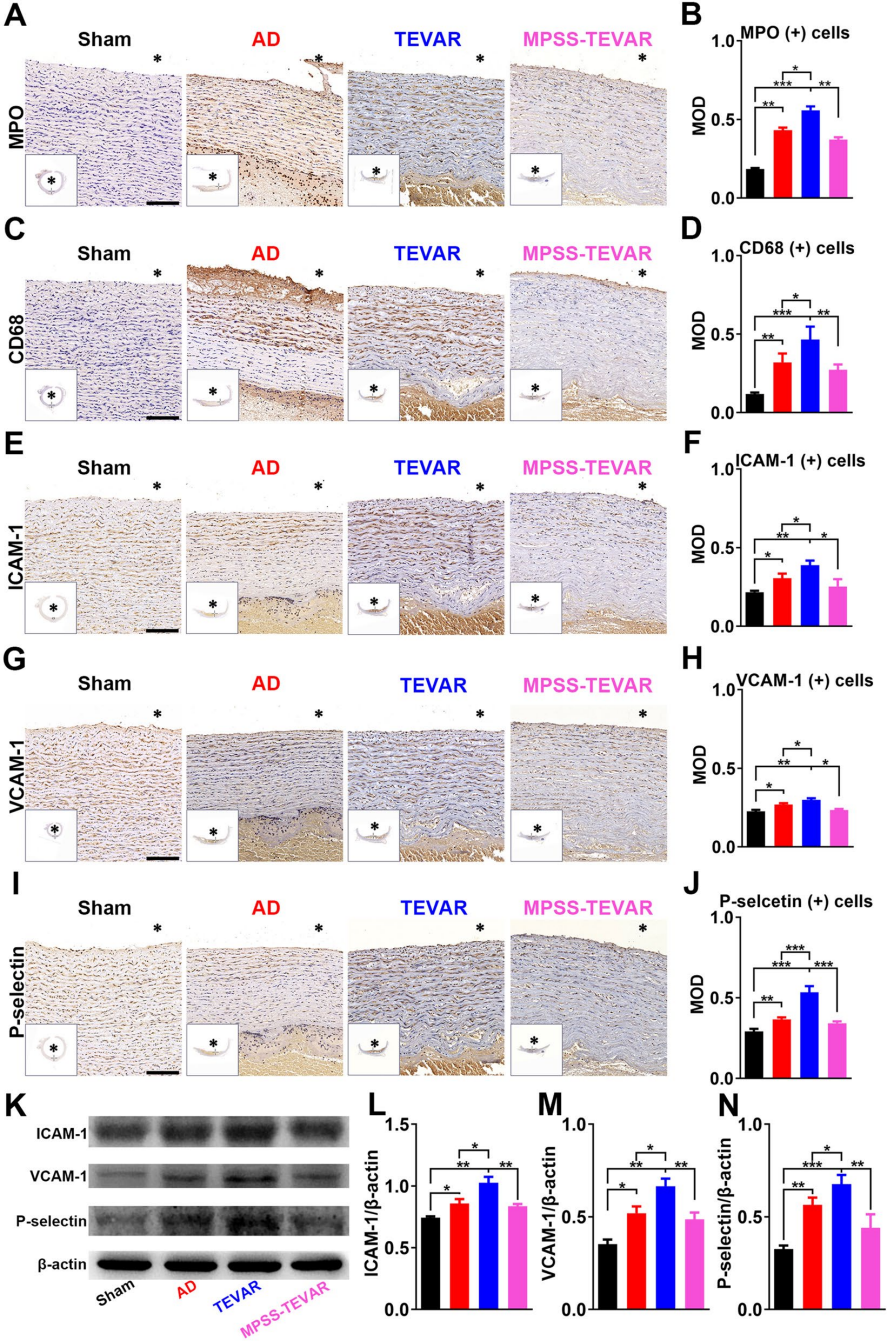
MPSS-loaded stent-grafts reduced inflammatory cells infiltration and expression of ICAM-1, VCAM-1 and P-selectin in endothelial cells. **A** Cell staining positive for MPO in each group, bar =100 μm; **B** Semi-quantitative analysis of MPO-positive cells in each group; **C** Cell staining positive for CD68 in each group, bar =100 μm; **D** Semi-quantitative analysis of CD68-positive cells in each group; **E** Cell staining positive for ICAM-1in each group, bar =100 μm; **F** Semi-quantitative analysis of ICAM-1-positive cells in each group. **G** Cell staining positive for VCAM-1 in each group, bar =100 μm; **H** Semi-quantitative analysis of VCAM-1-positive cells in each group. **I** Cell staining positive for P-selectin in each group, bar =100 μm; **J** Semi-quantitative analysis of P-selectin-positive cells in each group; **K** Western blotting of porcine aortic tissues for the relative expression of ICAM-1, VCAM-1 and P-selectin in each group; **L**, **K** and **N** Semi-quantitative analyses of ICAM-1/β-actin, VCAM-1/β-actin and P-selectin/β-actin in each group. The data are expressed as mean ± SD. Statistical significance:* *P* < 0.05; ** *P* < 0.01; *** *P* < 0.001. MOD, mean optical density

Changes in aortic cellular constituents of MPO and CD68 were detected by IHC staining (Figs. 5A and C). In the TEVAR groups, MPO-positive cells and CD68-positive cells significantly increase, compared with the MPSS-TEVAR group (All *P* < 0.001 Fig. 5B and D). Figure 5C, E and G show the representative IHC images of ICAM-1, VCAM-1 and P-selectin in each group, respectively.

## Discussion

Stent-grafts have been used to treat aortic dissection since Dake et al. successfully conducted transluminal placement of endovascular stent-grafts in 13 patients three decades ago [17]. Traditional stent-grafts decrease the long-term risk of all-cause mortality in aortic dissection patients [18]. However, aortic dissection patients who receive endovascular repair may suffer from stent-graft-related adverse events, in which inflammation is confirmed to play an important role [5]. The membrane artificially covered on the stent-grafts was to exclude the entry tears on the dissecting aorta. However, clinical evidence showed that expanded-polytetrafluoroethylene (e-PTFE), one kind of stent-covered membranes, may cause more severe inflammatory response in patients after TEVAR [13, 19]. It was found that glucocorticoids administration after TEVAR could reduce inflammatory response [13]. However, prolonged infusion of glucocorticoids may reduce host resistance and increase the risk of infection. In this condition, a film featured with high drug loading, sustained release and anti-inflammatory effects was conceived and fabricated in this study.

In this research, PLCL was fabricated with electrospinning to mimic the morphology and structure of the native extracellular matrix and provide a suitable micro-environment for endothelial cell attachment and proliferation. Furthermore, PLCL was used to form the shell fiber component with MPSS being encapsulated for the purpose of sustained drug release. The release profiles demonstrated a quick release of MPSS during the initial release period of 5 days, followed by a period of slower, sustained release (Fig. 1F).

As demonstrated by previous studies [10, 11, 20], endothelial dysfunction leads to inflammation, apoptosis and degradation of ECM. To evaluate the potential cytotoxicity of MPSS-loaded films directly in contact with endothelial cells, cell prolieration study was conducted before in vivo examination. Results showed that MPSS-loaded films did not influence cell proliferation in vitro experiment.

To ensure the anti-inflammatory effect of MPSS-loaded stent-grafts, a porcine AD model was established with transluminal implantation of stent-grafts. The results from serum cytokine levels and PET-CT indicated that MPSS-loaded stent-grafts achieved a satisfactory anti-inflammatory effect. Although aortic inflammation was confirmed to be an independent risk factor of rupture, there were few therapeutic interventions have been proposed to date. To our best knowledge, this is the first study to demonstrate a reduction in local and systemic inflammation in AD by MPSS-loaded films applied to the abluminal stent graft surface.

To further confirm whether the reduced production of cytokines was related with inhibition of inflammation in dissecting aorta, aortic tissues were harvested for examinations. Result of IHC staining indicates higher expression of ICAM-1, VCAM-1 and P-selectin in TEVAR group than that of the AD group (Figs. 5E, G and I), which mediate leukocytes rolling and attaching on platelets. Previous study demonstrated that dexamethasone could reduce adhesion molecule expression over endothelial cells in vitro experiment. In our study, MPSS-loaded stent-grafts significantly reduced expression of ICAM-1, VCAM-1 and P-selectin, compared with TEVAR group (Figs. 5E, G and I). Once inflammatory cells infiltrate the dissecting aorta, followed by the degradation of ECM [20].

## Limitation

There is lack of an in vivo test evaluate the pharmacokinetic levels of MPSS released into surrounding tissue. Further studies are warranted to verify the ability of MPSS-loaded stent-grafts to perform controllable drug release in physiological environments.

## Conclusion

In summary, we developed an MPSS-loaded stent-graft fabricated with electronspining for the controllable drug release. In vitro cell coculture confirmed the biocompability. Furthermore, in vivo examination showed that MPSS-loaded stent-grafts improved inflammatory response and reduced degradation of ECM. This study showed that MPSS-loaded stent-grafts have the potential to improve the results of stent-graft treatment of AD by reducing the local and systemic inflammation and thereby potentially improve the long-term clinical outcomes.

## Supplementary Information

**Additional file 1: Supplemental Figure 1.** Porcine aortic dissection model and stent-graft implantation. (A) The thoracic aorta was exposed; (B) the adventitial and media of aorta was mechanically separated; (C) pulse-type injection of saline to make the dissection extend to distal aorta; (D) when the model was developed, the adventitial was sutured; (E) the intra-operative ultrasound imaging showed the entry, false lumen (FL) and true lumen (TL) of aortic dissection; (F) the reconstruction of porcine aortic dissection; (G) the reconstruction of porcine aortic dissection after stent-graft implantation; (H) the tissue sampling of aortic dissection after stent-graft implantation.

## Data Availability

The datasets used and/or analyzed during the current study are available from the corresponding author on reasonable request.
