# Supplementary figures and images for: A methylprednisolone-loaded and core-shell nanofiber-covered stent-graft to prevent inflammation and reduce degradation in aortic dissection
Source: Biomater Res. 2022 Apr 25;26:15. doi: 10.1186/s40824-022-00259-5 (PMC9036796; doi:10.1186/s40824-022-00259-5)

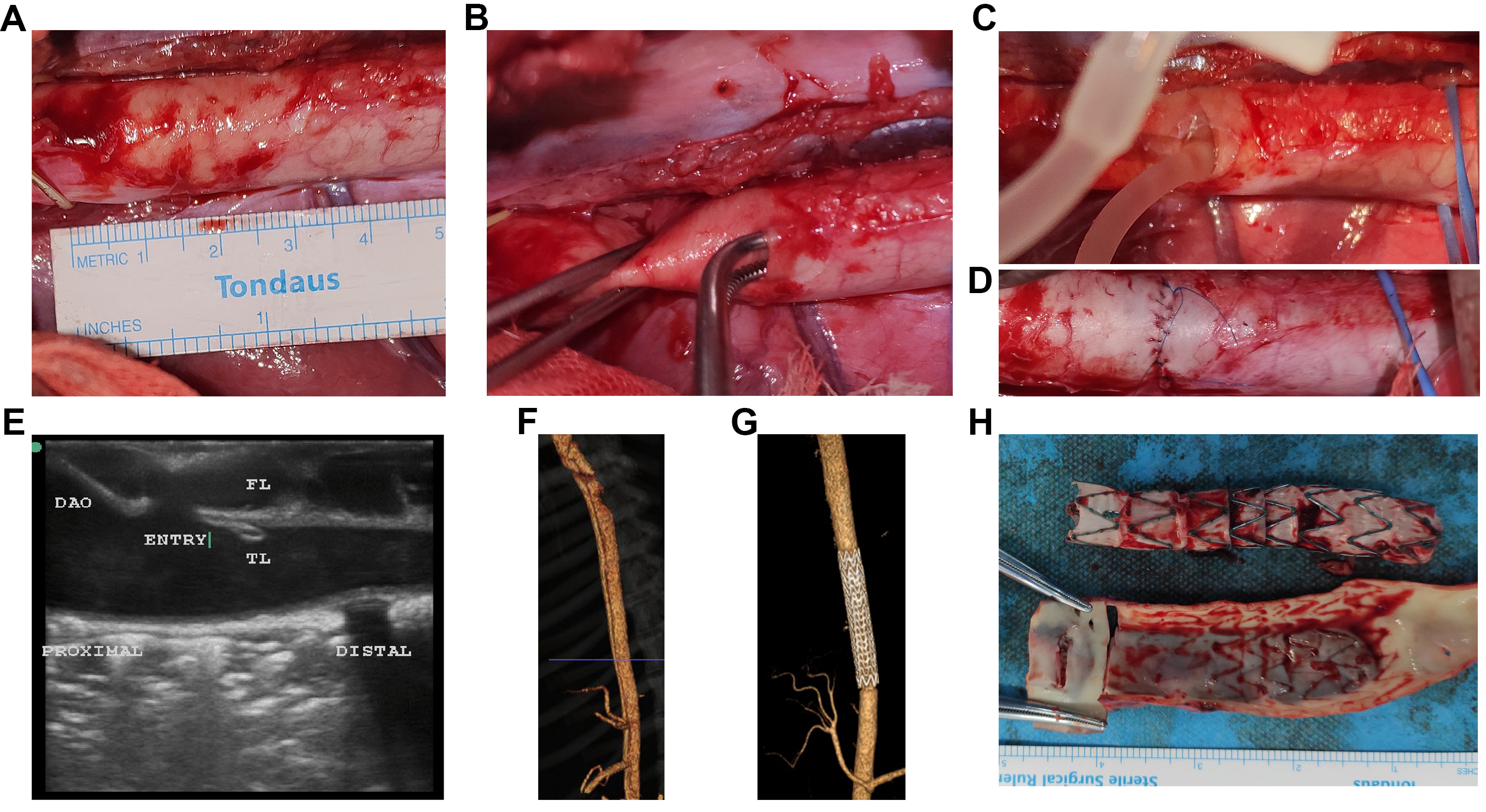

Supplement: Supplementary file 1 — Additional file 1: Supplemental Figure 1. Porcine aortic dissection model and stent-graft implantation. (A) The thoracic aorta was exposed; (B) the adventitial and media of aorta was mechanically separated; (C) pulse-type injection of saline to make the dissection extend to distal aorta; (D) when the model was developed, the adventitial was sutured; (E) the intra-operative ultrasound imaging showed the entry, false lumen (FL) and true lumen (TL) of aortic dissection; (F) the reconstruction of porcine aortic dissection; (G) the reconstruction of porcine aortic dissection after stent-graft implantation; (H) the tissue sampling of aortic dissection after stent-graft implantation. [file 40824_2022_259_MOESM1_ESM.jpg]
